# Comprehensive Analysis of RNA Expression Profile Identifies Hub miRNA-circRNA Interaction Networks in the Hypoxic Ischemic Encephalopathy

**DOI:** 10.1155/2021/6015473

**Published:** 2021-09-21

**Authors:** Lin Wei, Xia Li, Lijuan Wang, Yanyan Song, Hongmei Dong

**Affiliations:** ^1^Department of Neonatology, Jinan Maternal and Child Health Care Hospital, Shandong, China; ^2^Department of Pediatrics, Jinan Maternal and Child Health Care Hospital, Shandong, China; ^3^Department of Pediatrics, Chinese People's Liberation Army of 960 Hospital, Shandong, China

## Abstract

Hypoxic ischemic encephalopathy (HIE) is classified as a sort of serious nervous system syndrome that occurs in the early life period. Noncoding RNAs had been confirmed to have crucial roles in human diseases. So far, there were few systematical and comprehensive studies towards the expression profile of RNAs in the brain after hypoxia ischemia. In this study, 31 differentially expressed microRNAs (miRNAs) with upregulation were identified. In addition, 5512 differentially expressed mRNAs, long noncoding RNAs (lncRNAs), and circular RNAs (circRNAs) were identified in HIE groups. Bioinformatics analysis showed these circRNAs and mRNAs were significantly enriched in regulation of leukocyte activation, response to virus, and neutrophil degranulation. Pathway and its related gene network analysis indicated that HLA − DPA1, HLA − DQA2, HLA − DQB1, and HLA − DRB4 have a more crucial role in HIE. Finally, miRNA-circRNA-mRNA interaction network analysis was also performed to identify hub miRNAs and circRNAs. We found that miR-592 potentially targeting 5 circRNAs, thus affecting 15 mRNA expressions in HIR. hsa_circ_0068397 and hsa_circ_0045698 were identified as hub circRNAs in HIE. Collectively, using RNA-seq, bioinformatics analysis, and circRNA/miRNA interaction prediction, we systematically investigated the differentially expressed RNAs in HIE, which could give a new hint of understanding the pathogenesis of HIE.

## 1. Introduction

Hypoxic ischemic encephalopathy (HIE) is classified as a sort of serious nervous system syndrome that occurs in the early life period arising from placental insufficiency or umbilical cord obstruction in the perinatal period [1]. HIE is thought to be the chief neuro-developmental disorder in infants, with an estimated incidence rate of 1 to 6 per 1000 births [2, 3]. It has shown that HIE constitutes a primary inducer of incidence and lethality rates in neonates [4]. Afterwards, cell damage in the central nervous system caused by neonatal hypoxia and ischemia is still the common contributor to neurological disorders in adulthood, such as cerebral palsy and mental retardation [1–5]. Despite mounting studies have revealed the morphological, biophysical, and biochemical changes that occur after neonatal hypoxia ischemia (HI) brain damage, the efficacy of neuro-protective treatments is limited to HIE patients [6]. In order to give impetus to neurotherapeutics and ameliorate clinical outcomes in HIE, powerful biomarkers towards brain injury are essential to quickly assess the effect of treatment, offer prognostic details for family members, and guide the needs of later recovery care, thereby contributing to personalized care [6, 7].

Currently, miRNAs have attracted the attention of many researchers due to their crucial roles in posttranscriptional regulation in human diseases. There are several studies that have shown most miRNAs are observed in brain tissues. Unique properties of miRNAs as potential markers for a variety of diseases, such as oncology and heat disease [8–10]. Additionally, circular RNA (CircRNA) has been confirmed to participate in many biological functions, comprising the promotion of rolling circle translation, the control of parent genes transcription, the assistant of alternatively spliced mRNA formation, and the sponging actor of microRNA (miRNA/miR) [11, 12]. It has been shown that changes in the levels of specific circRNAs exhibited an association with carcinoma, ischemia, stroke, neurodegenerative diseases, heart disease, etc. [13, 14]. CircRNAs, especially in brain tissue, were not only related to the nervous system's development, differentiation, and biological function but also functioned importantly in the dysfunction and pathology after brain damage [15]. Recently, it has been reported that circRNAs' expression profile in brain tissue was modified under cerebral ischemia, among which were probably involved in the etiopathogenesis of cerebral ischemia. In adult rats, there were some studies [11, 16] showing that the expression profiles of cerebral circRNA altered significantly following stroke, which was conducive to stabilizing mRNA expression. Also, several circRNAs had been revealed to be related to HIE. For example, circular RNA cZNF292 silence alleviates neural injury through upregulation of miR-22 in rat model [17]. In addition, Jiang et al. identified a total of 66 circRNAs were differentially expressed in HIBD rats compared with the control group [18]. So far, there were few systematical and comprehensive studies towards the expression profile of RNAs in the brain after hypoxia ischemia.

In our study, we attempted to investigate novel disease-related RNAs in HIE–induced rat model to decipher the biological function of RNAs. RNA sequencing (RNA-seq) is a powerful tool for analyzing the changes of transcriptome in cells and tissues [19]. This method can obtain information related to RNA splicing, gene expression, and single nucleotide polymorphism (SNP) change [19]. It can also be utilized as a distinguishing tool for ribosomal RNA, transfer RNAs, microRNAs, and small RNAs. In recent years, RNA-seq has been largely applied to identify the differential expression of genes. Herein, the expression profiles of RNAs were analyzed a by RNA-seq. The possible function of RNAs in HIE was predicted by gene ontology (GO) and Kyoto Encyclopedia of Genes and Genomes (KEGG) pathway analysis. Furthermore, miRNAs regulated by differentially expressed RNAs were predicted in TargetScan database. Collectively, the combined use of RNA-seq data and bioinformatics analysis in out literature uncovered the potential roles of these crucial RNAs in pathophysiological processes of HIE.

## 2. Materials and Methods

### 2.1. Sample Collection

The two RNA-seq dataset and the corresponding metadata were acquired from Genes Expression Omnibus (GEO) database (http://www.ncbi.nlm.nih.gov/geo) database (GSE164727 and GSE121178 [20]). Three healthy neonatal rats (control group) and three HIBD-induced rats (experimental group) were distributed in GSE164727 dataset. Three whole blood samples from HIE-induced rats and three normal blood samples from control group were allocated in GSE121178 dataset [20].

### 2.2. Differential Expression Analysis

The count matrix of gene expression and the corresponding grouping data were obtained from GEO database and converted to count-per-million (CPM) for difference analysis. The limma package in the R software (version 3.3.2, https://www.r-project.org/) was utilized to screen the differentially expressed miRNAs and circRNAs, with the selection criterion of adjusted *p* value < 0.05 and ∣log2 fold change (FC) | >1. The DEGs with log2 FC < −1 were DEGs with downregulation, whereas those with log2 FC > 1 were DEGs with upregulation.

### 2.3. Enrichment Analysis

Gene Ontology (GO), KEGG pathway analysis, and Reactome pathway analysis were performed. Hypergeometric distribution was applied to calculate the *p* value. R package “clusterProfiler” (version 3.3.2) was employed to conduct all enrichment analysis [28]. *p* < 0.05 meant there was greatly significant difference in two or more compared groups.

### 2.4. Gene Set Enrichment Analysis (GSEA)

GSEA is taken to determine whether a set of a priori defined genes display significant statistics differences in two biological states (e.g., phenotypes). GSEA was conducted by the GSEA software (version 1.46.0) [21].

### 2.5. Network Analysis

The parent genes of the differentially expressed circRNAs and their loci in the genome were searched in the circBase database (version 1.0 http://www.circbase.org/) [22]. These circRNAs were then searched in the CSCD database (version 1.0 http://gb.whu.edu.cn/CSCD) [23], and the corresponding data was utilized to plot circRNA circle diagrams. TargetScan (version 7.1 http://www.targetscan.org/vert_71/) was applied to predict miRNAs' binding sites. Finally, the network is visualized by the Cytoscape software (version 3.8.2) [24].

### 2.6. Statistical Analysis

R (version 3.6.1) was applied for statistical analysis, and Wilcoxon rank sum test was utilized to investigate the difference between the two groups. *p* < 0.05 meant that significantly statistics difference existed in compared groups. The pheat map package (version 1.0.12) was applied to plot the heat map for the DEGs. The R package “ggbiplot” (version 0.55) was employed to conduct the principal component analysis (PCA) for miRNAs.

## 3. Results

### 3.1. Identification and Characteristic Comparisons of miRNAs, circRNAs, and mRNAs

**Figure 1:**
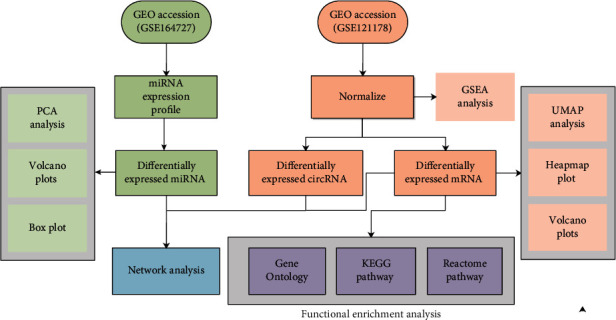
The flow chart analysis of this manuscript.

**Figure 2:**
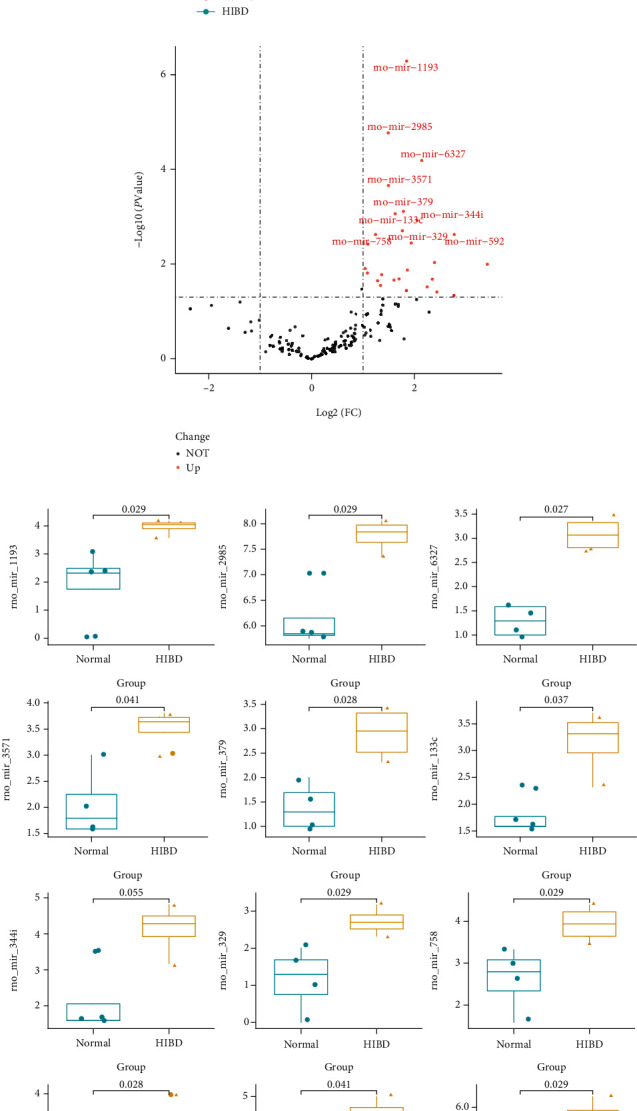
Differentially expressed miRNAs between hypoxic ischemic brain damage (HIBD) and normal group. (a) PCA analysis for all the miRNAs. (b) Volcano plot for differentially expressed miRNAs (*p* < 0.05); the red dots represent the upregulated in model rat group; black dots mean there is no significant difference between the two groups. (c) Boxplot for the most top 12 miRNAs (*p* < 0.05), including rno_mir_1193, rno_mir_133c, rno_mir_186, rno_mir_2985, rno_mir_329, rno_mir_344i, rno_mir_3571, rno_mir_379, rno_mir_592, rno_mir_6327, rno_mir_679, and rno_mir_758.

The flow chart analysis is illustrated in Figure 1. Totally, 627 miRNAs were identified. After excluding miRNAs with low expression (mean expression < 1), 179 miRNAs were retained for subsequent screening and applied for PCA analysis.  Figure 2(a) shows that the majority of samples in the same group were classified into the same group, indicating that the miRNA expression between model mice and normal mice was significantly different. 31 differentially expressed miRNAs with upregulation were identified in HIBD/HIE rats group after comparison with the normal group (Figure 2(b)). We further evaluated the expression value for whole samples with the differentially expressed miRNAs between the two groups, and the most top 12 differentially expressed miRNAs were chosen to construct a boxplot, including rno_mir_1193, rno_mir_133c, rno_mir_186, rno_mir_2985, rno_mir_329, rno_mir_344i, rno_mir_3571, rno_mir_379, rno_mir_592, rno_mir_6327, rno_mir_679, and rno_mir_758 (Figure 2(c)).

**Figure 3:**
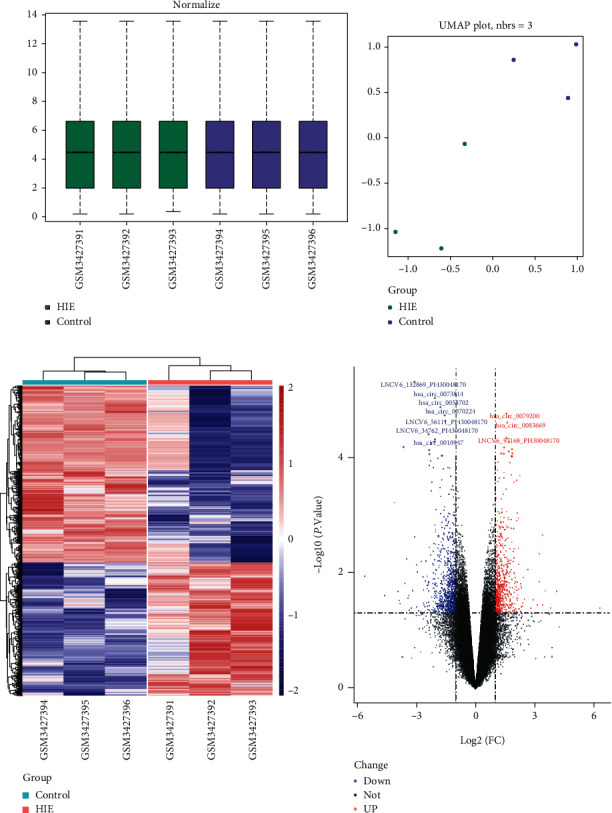
Differentially expressed RNAs between hypoxic ischemic encephalopathy (HIE) and normal group. (a) Boxplot for sample normalization display. (b) UMAP plot for all the RNAs. (c) Heat map plot for all the differentially RNAs; red indicates high expression, and blue is opposite. (d) Volcano plot for differentially expressed RNAs (*p* < 0.05); the red dots represent the upregulated in HIE group, blue dots represent the downregulated in HIE group, and black dots mean there is no significant difference between the two groups.

Next, we normalized the expression profile data (Figure 3(a)). The UMAP plot shows that the HIE group and normal group have different expression patterns (Figure 3(b)). A total of 5512 differentially expressed mRNAs, lncRNAs, and circRNAs were identified in the experimental and control groups. Among them, 2363 genes were upregulated compare the HIE group to the normal group, and 3149 genes were downregulated (Figure 3(c)). Then, we normalize the expression values of these 5512 genes and draw the heat map with cluster the samples. The results show that the samples in the identical group can be clustered together well (Figure 3(d)).

Among these different genes, we revealed 1188 upregulated circRNAs, 1244 downregulated circRNAs, 908 upregulated lncRNAs, 1514 downregulated circRNAs, 268 upregulated mRNAs, and 389 downregulated circRNAs in the HIE group compared to the control group. The top 10 upregulated and downregulated mRNAs, lncRNAs, and circRNAs are listed in Tables 1–3.

### 3.2. Functional Enrichment Analysis for All Differentially Expressed circRNAs and mRNAs

**Figure 4:**
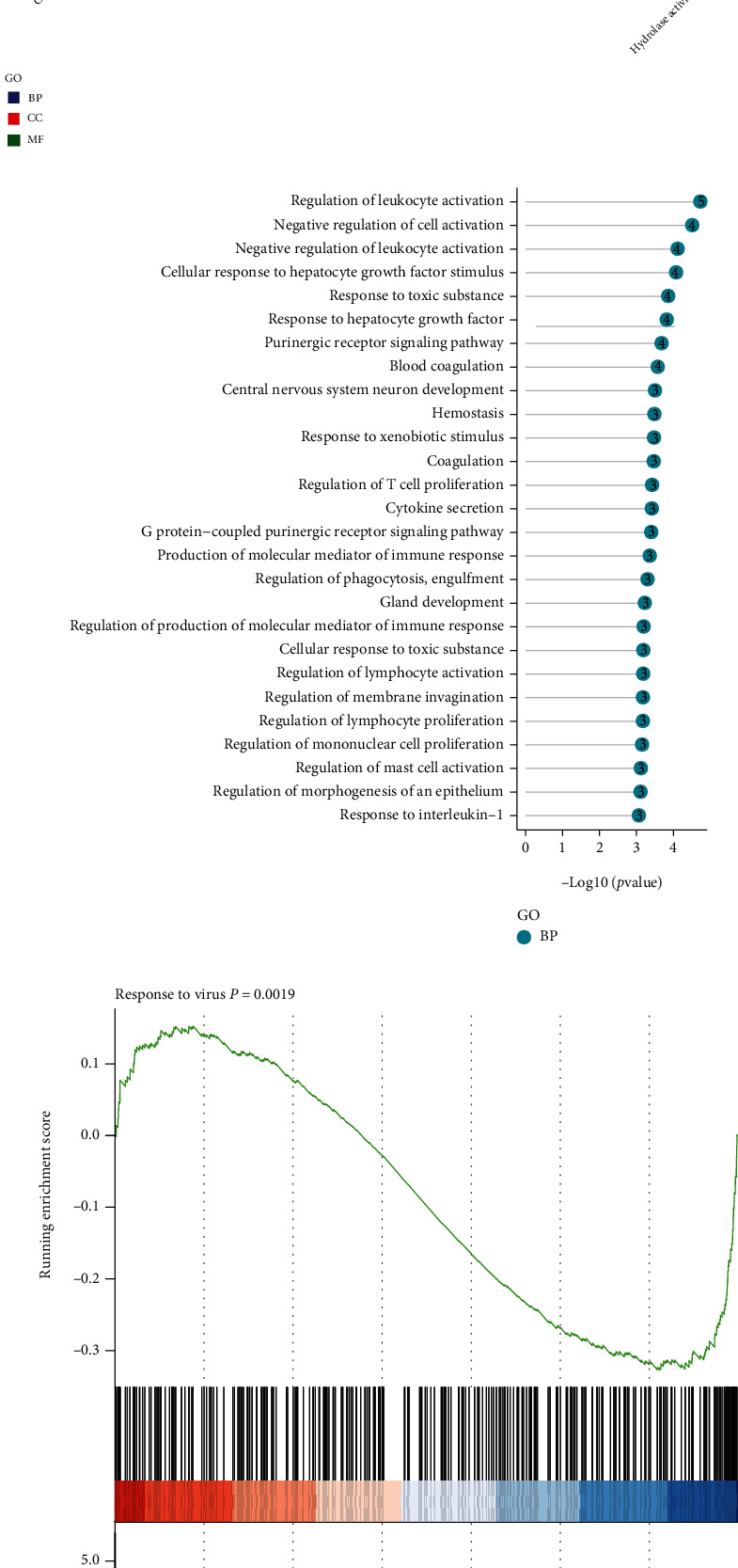
Functional enrichment analysis for differentially expressed RNAs. (a) Pathway analysis of differently expressed RNAs (*p* < 0.05). (b) The top correlated biological processed of differently expressed RNAs (*p* < 0.05). (c) The most significantly enriched term in Biological Process for GSEA (*p* < 0.05).

There are 29 GO terms that were enriched in the differentially expressed circRNAs and mRNAs which include 14 Cellular Component (CC) terms, 4 Biological Process (BP) terms, and 9 Molecular Function (MF) terms (Figure 4(a)). Among them, regulation of leukocyte activation, negative regulation of cell activation and leukocyte activation, and cellular response to hepatocyte growth factor stimulus were the most significantly enriched in BP (Figure 4(b)). The GSEA results showed that 136 GO terms were enriched for the differentially expressed RNAs. The most significantly enriched term in BP is response to virus (Figure 4(c)).

**Figure 5:**
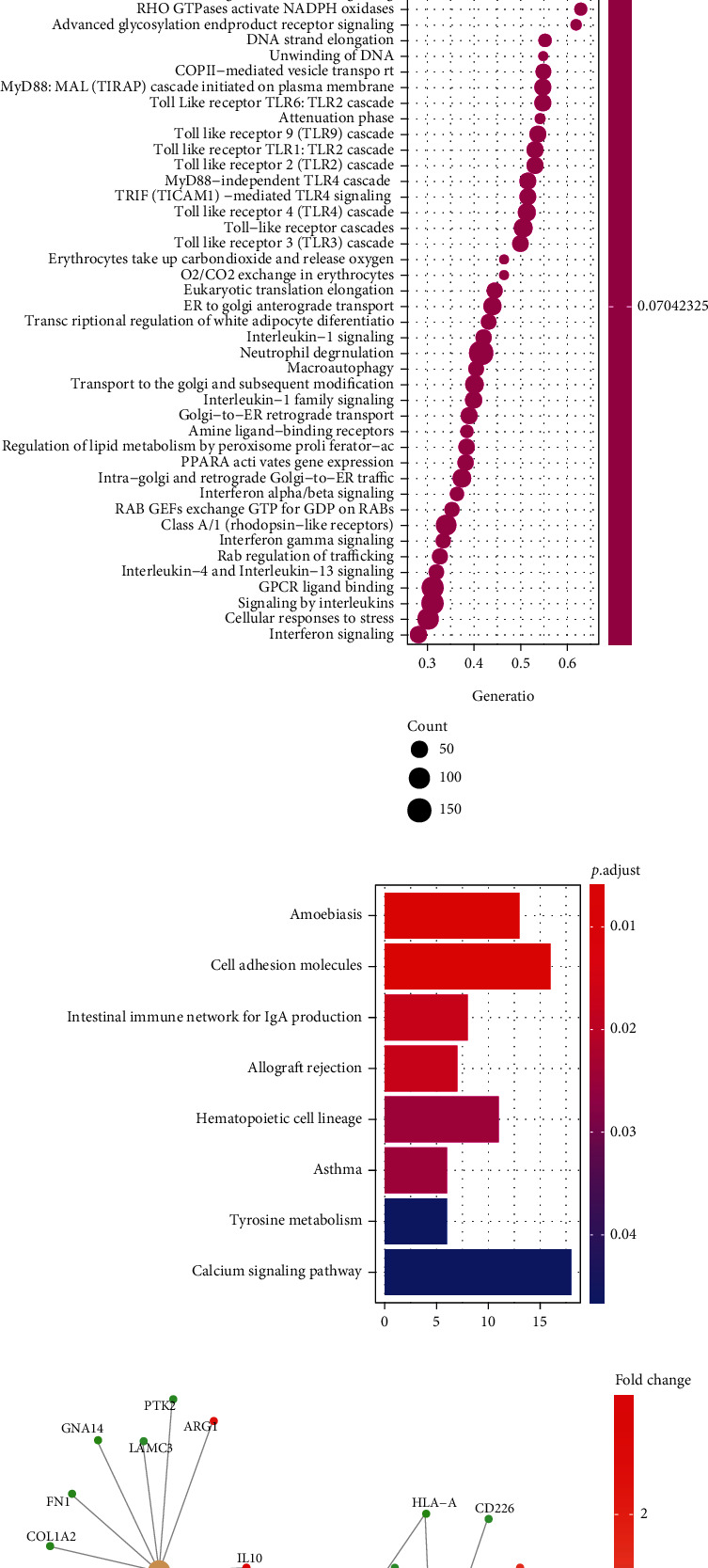
Pathway enrichment analysis for differentially expressed RNAs (DERNAs). (a) Significantly KEGG enriched terms in for DERNAs (*p* < 0.05). (b) Significantly enriched terms in Reactome for DERNAs (*p* < 0.05). (c) Enriched pathway in KEGG (*p* < 0.05). (d) Network for the enriched pathway in KEGG and related genes. (e) Differentially expressed genes in KEGG pathways (*p* < 0.05).

Additionally, there were 8 pathways in KEGG and 25 pathways in Reactome enriched for these genes, respectively (Figures 5(a) and 5(b)). And the pathways for amoebiasis, intestinal immune network for IgA production, allograft rejection, and hematopoietic cell lineage are the most enriched pathways in KEGG (Figures 5(c) and 5(e)). Pathway and its related genes network analysis indicated that the pathways with significant enrichment have a strong correlation (Figure 5(d)). Multiple pathways were potentially regulated by several hub genes; for example, ICOS, HLA − A, and CD40LG were related to regulate allograft rejection and cell adhesion molecules; IL10 was related to regulate allograft rejection, amoebiasis, and hematopoietic cell lineage (Figure 5(d)). Of note, we observed that HLA − DPA1, HLA − DQA2, HLA − DQB1, and HLA − DRB4 have a more crucial roles in HIE by modulating 8 pathways (Figure 5(e)).

### 3.3. miRNA-circRNA-mRNA Interaction Network Analysis

**Figure 6:**
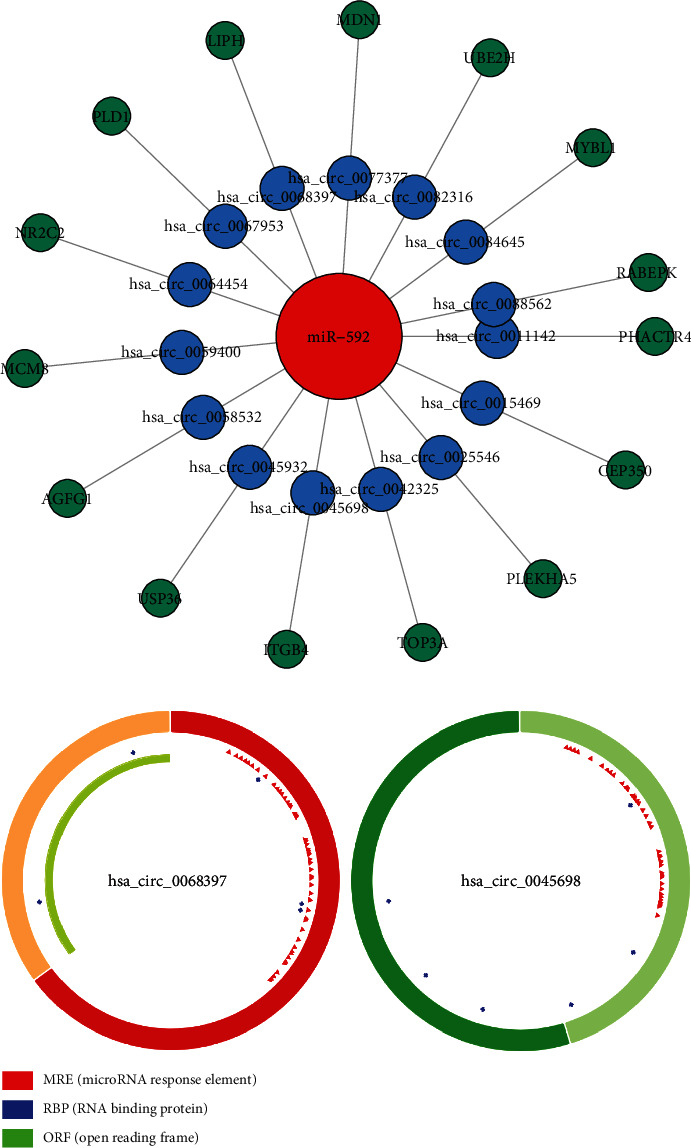
miRNA, circRNA, and mRNA interaction network. (a) miRNA-circRNA-mRNA interaction network analysis for miR-592. The red represents miRNA, blue represents circRNA, and line blue is the mRNA. (b) The circRNA analysis for the most significant circRNA (hsa_circ_0045698) and the circRNA (hsa_circ_0068397), which indicated the number and position of MRE (red), RBP (blue), and ORF (green) elements in circRNAs.

2432 differentially expressed circRNAs were identified. Among them, 772 have comments in CDC database. A total of 2055 miRNAs was predicted in TargetScan database. Then, we matched these predicted miRNAs with the miRNAs screened in the previous section, and the results showed that only miR-592 is available in both datasets. Through screening, we found that a total of 15 circRNA (hsa_circ_0011142, hsa_circ_0015469, hsa_circ_0025546, hsa_circ_0042325, hsa_circ_0045698, hsa_circ_0045932, hsa_circ_0058532, hsa_circ_0059400, hsa_circ_0064454, hsa_circ_0067953, hsa_circ_0068397, hsa_circ_0077377, hsa_circ_0082316, hsa_circ_0084645, hsa_circ_0088562) response elements contain miR-592, and these circRNAs are in different gene regions. And then, we used Cytoscape to draw the network for all miRNA, circRNAs, and mRNAs (Figure 6(a)). Among these circRNAs, the bioinformatics analysis demonstrated that hsa_circ_0045698 and hsa_circ_0068397 were the most significantly circRNAs, which including the most miRNA binding sites. As present in Figure 6(b), the CSCD database was used to draw a circle graph for these miRNAs of which targets are also differentially expressed (Figure 6(b)).

## 4. Discussion

HIE is a main contributor to higher incidence and fatality rates in neonates. Accompanied by the advancement of obstetrical and neonatal care, the survival probability of HIE has also heightened [1, 3, 25]. Nevertheless, the presently available treatment for therapeutic hypothermia can only benefit about 10% of babies. More importantly, it is still elusive towards the mechanisms of HIE. HIE is still considered as a detriment to children's health and life quality, and it is urgent to explore efficacious neurotherapeutic interventions and promising biomarker in the treatment of HIE [26, 27].

Until now, there are limited publicly reported studies on the expression of miRNAs in HIE. Shi et al. found that miRNA-21 and HIF-1*α* expression levels were higher in HIE neonate serum samples using a small cohort of newborns with HIE. Cai et al. showed that overexpression of miR-27a weakened HI-induced neuronal apoptosis via modulating FOXO1 in rat model [28]. Hypoxia-induced miR-152 suppresses cell apoptosis via inhibiting PTEN [29]. The present study determined the expression profile of differentially expressed miRNAs using RNA-Seq along with bioinformatics analysis. A total of 627 miRNAs were detected, 179 of which were chosen for PCA analysis. 31 differentially expressed miRNAs with upregulation were identified after comparing the two groups, and the most top 12 differentially expressed miRNAs, including rno-mir-1193, rno-mir-2985, rno-mir-6327, rno-mir-3571, rno-mir-379, rno-mir-344i, rno-mir-133c, rno-mir-329, rno-mir-758, and rno-mir-592. Among these miRNAs, miR-329-5p was found to interact with upregulated mmu-circRNA-015947 in neuron injury condition [18]. Mir-592 was reported to regulate the induction and cell death-promoting activity of p75NTR in neuronal ischemic injury [30]. miR-344 was reported neural-specific expressed during mouse embryonic development, indicated it had a crucial role in brain development [31]. Abnormal expression of mir-379 implies the pathogenesis of spinal cord injury [32]. These previous reports and this study indicated these miRNAs may play a key role in brain injury.

The microarray analysis also consisted of the expression profile of coding genes. We further evaluated the differentially expressed RNAs between HIE and normal groups. A total of 5512 differentially expressed mRNAs, lncRNAs, and circRNAs were identified in HIE and control groups. Among them, 2363 genes were induced, and 3149 genes were suppressed after comparison with the two groups. Typically, hsa_circ_0079200 and Hsa_circ_ 0083669 were significantly upregulated, while hsa_circ_0073814, hsa_circ_058702, and hsa_circ_0070224 were greatly downregulated. LNCV6_97168 was upregulated, whereas LNCV6_132869, LNCV6_56111, and LNCV6_34762 were downregulated. These results suggested that these altered lncRNAs and circRNAs in the pathophysiological processes following HIE could be potential biomarkers or promising therapeutic targets in treating this disease. GO analysis of these circRNAs and mRNAs was significantly enriched in regulation of leukocyte activation and cellular response to hepatocyte growth factor stimulus. The GSEA data revealed that there are 136 enriched GO terms for the differentially expressed RNAs, and the most significantly enriched biological process term is response to virus. KEGG analysis shows that amoebiasis, intestinal immune network for IgA production, allograft rejection, and hematopoietic cell lineage are the most enriched pathways. GSEA Reactome analysis indicated that neutrophil degranulation, signaling by interleukins, GPCR ligand binding, and cellular response to stress were the most significantly enriched terms for these RNAs.

Neutrophils are the first type of peripheral inflammatory cells in acute and chronic inflammation. Several lines of evidences have shown that infiltration of neutrophils in the adult cerebral ischemic area within a few hours after injury. In the neonatal HI injury model, the response of neutrophils in the brain is usually weakened because they seem to stay mainly in the blood vessels [33]. The expression of interleukin 1*β* (IL-1*β*) in cord blood and peripheral blood of HIE children was significantly upregulated, and the upregulated level was corresponding to HIE grades and adverse outcomes [34]. The release of IL-1*β* relied on the activation of NLRP3 inflammasome [35]. In disease models such as cerebral hemorrhage model and ischemia-reperfusion injury model, NLRP3 inflammasome was reported to be motivated in astrocytes [36, 37]. Mitochondria are the main target of HI injury. The impairment of mitochondrial increased with age, bringing about the disorder of mitochondrial-related molecular pathways. During HI injury, reactive oxygen species (ROS) generated by mitochondria exceeded the antioxidant capacity, resulting in DNA damage, mitochondrial lipid peroxidation, and Ca2+ homeostasis disruption. Therefore, mitochondrial function played as key determinant of survival during ischemic injury [38].

Multiple genes were dramatically upregulated in neonatal HIE, including IL10, ARG1, and IL1R2. Interleukin 10 (IL-10) is a synthetic human cytokine inhibitor with immunomodulatory and anti-inflammatory effects. It is the key to successfully resist the damage of host tissue during the acute phase of immune responses [39]. A large number of studies have verified the changes of IL-10 as well as its important role in HI injury, indicating that the release of IL-10 was increased, and it had a negative correlation with the rate of neuronal apoptosis [46]. For instance, Bai et al. [39] reported that the level of IL-10 was markedly raised after HI and IL-10 were colocalized with HI insult-injured neurons and astrocytes. IL-10 was shown to exhibit a repressive effect on immune responses and a decreased inflammatory reactions along with neuronal injury [40] in cortical tissues [40] and in serum cytokines of the liver [40]. Despite the expression of IL-10 was largely studied, the mechanism of IL-10 involving in HIE ignition and progression was still elusive [41]. Arginases are pivotal modulatory enzymes of inflammation and tissue repair [42]. At present, it is reported that the expression of ARG-1 in the blood of patients with stroke is heightened, and its level is related to infarct size [43], implying the involvement of ARG in the mechanisms of post-hypoxic-ischemia. Till now, there were emerging researches that have showed that ARG-1 was primarily related to the role of an indicator of “pro-repair” microglia, while ARG-2 was mainly related to cerebral blood flow regulation [44]. Hypoxia and ischemia-reperfusion injury are considered to be powerful stimuli for the expression of ARG-1 and ARG-2. IL1R2 is a cytokine receptor of the interleukin-1 (IL-1) receptor family, and it plays as an essential mediator of several cytokines generated by immune and inflammatory responses. Highly expressed IL1R2 was positively correlated with IL1R signals, manifesting that IL1R2 may participated in the interplay between IL1R1 and IL-1. In hypoxia/reoxygenation- (H/R-) treated cardiomyocytes, overexpressing IL1R2 attenuated the promoting proliferation and antiapoptosis effects induced by the overexpression of LncRNA A2M AS1 [45].

It has been reported that circRNAs act as a miRNA sponge and inhibit miRNA activity, leading to the upregulation of miRNA targets [46]. miRNAs had a crucial role in the central nervous system. Some circRNAs may took part in HIBD development through circRNA/miRNA interactions. miRNA target prediction software was utilized to prove that the dysregulated circRNAs that included miRNA binding sites [47]. A total of 2,432 differentially expressed circRNAs were identified, amid which 772 were annotated in CDC database. 2055 miRNAs were predicted in TargetScan database, and we found that only miR-592 is available in both datasets. Moreover, many previous studies had demonstrated that miR-592 was related to multiple nervous disorders [48–50]. A previous report has identified region-specific differences in miR-592 levels in the brain of adult rodent. Additionally, some reports have presented that miR-592 was differentially expressed in multiple neoplasms. One study showed that the altered miR-592 exerted impressive consequences in the nervous system by modulating the expression of p75NTR [30].

Finally, we found the most significantly differentially expressed circRNAs (hsa_circ_0045698 and hsa_circ_0068397). Future researches will concentrate on exploring more downstream targets of both two circRNAs, helping to decipher their role in the pathophysiological process of neonatal HIBD. Collectively, using RNA-seq, bioinformatics analysis, and circRNA/miRNA interaction prediction, we systematically investigated the differentially expressed RNAs in HIE model. These discoveries could give a new hint of understanding the pathogenesis of HIE and in depth deciphering the biological functions of these candidate circRNAs/miRNAs.

## Figures and Tables

**Table 1:** The top 10 upregulated and downregulated mRNAs.

ID	Accession	logFC	AveExpr	P.Value
LNCV6_137283_PI430048170	NM_001290053	-4.5712	9.1133	0.0249
LNCV6_143249_PI430048170	NM_021983	-3.9035	10.6029	0.0210
LNCV6_132172_PI430048170	NM_020386	-3.6672	2.4669	0.0001
LNCV6_130591_PI430048170	NM_017636	-3.6319	4.3296	0.0045
LNCV6_129873_PI430048170	NM_001140	-3.4577	6.2115	0.0058
LNCV6_141748_PI430048170	NM_001005213	-3.3338	9.6179	0.0332
LNCV6_132869_PI430048170	NM_001040274	-3.1152	2.0956	0.0000
LNCV6_144769_PI430048170	NM_000062	-2.9203	9.9406	0.0322
LNCV6_128219_PI430048170	NM_001870	-2.8884	4.7753	0.0025
LNCV6_66130_PI430048170	NM_001032392	-2.7465	10.1516	0.0304
LNCV6_131968_PI430048170	NM_001146705	2.7202	4.8168	0.0367
LNCV6_131015_PI430048170	NM_001297650	2.7504	2.1087	0.0233
LNCV6_128757_PI430048170	NM_001657	2.7809	2.1793	0.0412
LNCV6_130521_PI430048170	NM_001145777	3.0223	2.4875	0.0104
LNCV6_138042_PI430048170	NM_020927	3.1912	1.9191	0.0018
LNCV6_46971_PI430048170	NM_018324	3.2760	1.9546	0.0432
LNCV6_137605_PI430048170	NM_000572	3.3804	1.9115	0.0364
LNCV6_130004_PI430048170	NM_004666	3.6646	9.0162	0.0176
LNCV6_134373_PI430048170	NM_004633	3.7335	12.5614	0.0411
LNCV6_129250_PI430048170	NM_004681	6.2711	3.5478	0.0409

**Table 2:** The top 10 upregulated and downregulated lncRNAs.

ID	Accession	logFC	AveExpr	P.Value
LNCV6_120464_PI430048170	NONHSAT044111	-5.6589	3.2338	0.0208
LNCV6_95437_PI430048170	NR_001298	-5.5896	8.6021	0.0115
LNCV6_39456_PI430048170	lnc-CLMP-7 : 1	-4.3486	9.2576	0.0334
LNCV6_124812_PI430048170	NONHSAT118504	-4.0876	6.8167	0.0006
LNCV6_88211_PI430048170	lnc-CBR3-2 : 1	-3.9918	3.5952	0.0015
LNCV6_12711_PI430048170	ENST00000587085	-3.8426	9.3581	0.0304
LNCV6_75832_PI430048170	lnc-GIN1-6 : 1	-3.7582	4.7057	0.0292
LNCV6_67231_PI430048170	lnc-EPSTI1-4 : 2	-3.5581	3.2978	0.0153
LNCV6_90811_PI430048170	lnc-ANKRD30A-5 : 4	-3.4008	6.2422	0.0279
LNCV6_16175_PI430048170	ENST00000504301	-3.3544	3.9316	0.0252
LNCV6_53935_PI430048170	NR_033298	2.5849	3.2311	0.0124
LNCV6_99652_PI430048170	NR_027034	2.6022	3.8056	0.0491
LNCV6_103450_PI430048170	NR_130928	2.6339	8.9807	0.0089
LNCV6_31834_PI430048170	ENST00000583578	2.7676	2.0526	0.0071
LNCV6_110534_PI430048170	lnc-PRKCE-2 : 1	2.8019	1.6291	0.0012
LNCV6_57143_PI430048170	lnc-HBD-1 : 1	3.3394	4.2881	0.0129
LNCV6_35200_PI430048170	NR_001545	3.3749	3.7568	0.0023
LNCV6_106991_PI430048170	lnc-TSEN2-4 : 1	3.5024	3.2133	0.0207
LNCV6_83797_PI430048170	NR_045129	3.7757	2.3294	0.0410
LNCV6_32804_PI430048170	ENST00000619715	5.3539	3.0591	0.0282

**Table 3:** The top 10 upregulated and downregulated circRNAs.

ID	Accession	logFC	AveExpr	P.Value
hsa_circ_0043393	hsa_circ_0043393	-4.7357	8.2172	0.0218
hsa_circ_0091074	hsa_circ_0091074	-4.7143	4.8620	0.0412
hsa_circ_0091073	hsa_circ_0091073	-4.6824	4.9597	0.0305
hsa_circ_0007362	hsa_circ_0007362	-4.0599	9.3619	0.0443
hsa_circ_0078345	hsa_circ_0078345	-3.9914	9.4687	0.0370
hsa_circ_0050202	hsa_circ_0050202	-3.7979	8.8866	0.0344
hsa_circ_0020789	hsa_circ_0020789	-3.7876	6.2214	0.0330
hsa_circ_0057101	hsa_circ_0057101	-3.6401	8.8868	0.0219
hsa_circ_0066142	hsa_circ_0066142	-3.5134	9.2071	0.0267
hsa_circ_0038336	hsa_circ_0038336	-3.4186	8.6167	0.0463
hsa_circ_0060880	hsa_circ_0060880	3.1128	2.8186	0.0366
hsa_circ_0080915	hsa_circ_0080915	3.2046	3.4014	0.0106
hsa_circ_0003368	hsa_circ_0003368	3.2062	1.8505	0.0048
hsa_circ_0029023	hsa_circ_0029023	3.2249	2.1885	0.0144
hsa_circ_0075950	hsa_circ_0075950	3.2634	1.8496	0.0027
hsa_circ_0080914	hsa_circ_0080914	3.6354	3.0716	0.0193
hsa_circ_0069997	hsa_circ_0069997	3.6582	3.2195	0.0267
hsa_circ_0080916	hsa_circ_0080916	3.7093	2.8606	0.0121
hsa_circ_0092220	hsa_circ_0092220	4.0974	4.6393	0.0210
hsa_circ_0092222	hsa_circ_0092222	6.5836	4.4162	0.0418

## Data Availability

The data used during the present study are available from the corresponding author upon reasonable request.

## References

[1] Xiong L. L., Xue L. L., Al-Hawwas M. (2020). Single-nucleotide polymorphism screening and RNA sequencing of key messenger RNAs associated with neonatal hypoxic-ischemia brain damage. *Neural Regeneration Research*.

[2] Chau V., Poskitt K. J., Dunham C. P., Hendson G., Miller S. P. (2014). Magnetic resonance imaging in the encephalopathic term newborn. *Current Pediatric Reviews*.

[3] Vannucci R. C. (2000). Hypoxic-ischemic encephalopathy. *American Journal of Perinatology*.

[4] Wachtel E. V., Hendricks-Munoz K. D. (2011). Current management of the infant who presents with neonatal encephalopathy. *Current Problems in Pediatric and Adolescent Health Care*.

[5] Shankaran S., Laptook A. R., Ehrenkranz R. A. (2005). Whole-body hypothermia for neonates with hypoxic-ischemic encephalopathy. *The New England Journal of Medicine*.

[6] Massaro A. N., Wu Y. W., Bammler T. K. (2018). Plasma biomarkers of brain injury in neonatal hypoxic-ischemic encephalopathy. *The Journal of pediatrics*.

[7] Bersani I., Piersigilli F., Gazzolo D. (2021). Heart rate variability as possible marker of brain damage in neonates with hypoxic ischemic encephalopathy: a systematic review. *European Journal of Pediatrics*.

[8] Ashwal-Fluss R., Meyer M., Pamudurti N. R. (2014). circRNA biogenesis competes with pre-mRNA splicing. *Molecular Cell*.

[9] Ponnusamy V., Yip P. K. (2019). The role of microRNAs in newborn brain development and hypoxic ischaemic encephalopathy. *Neuropharmacology*.

[10] Wan X., Kong Z., Chu K. (2018). Co-expression analysis revealed PTCH1-3'UTR promoted cell migration and invasion by activating miR-101-3p/SLC39A6 axis in non-small cell lung cancer: implicating the novel function of PTCH1. *Oncotarget*.

[11] Rybak-Wolf A., Stottmeister C., Glazar P. (2015). Circular RNAs in the mammalian brain are highly abundant, conserved, and dynamically expressed. *Molecular Cell*.

[12] Li X., Yang L., Chen L. L. (2018). The biogenesis, functions, and challenges of circular RNAs. *Molecular Cell*.

[13] Mehta S. L., Pandi G., Vemuganti R. (2017). Circular RNA expression profiles alter significantly in mouse brain after transient focal ischemia. *Stroke*.

[14] Tang M., Kui L., Lu G., Chen W. (2020). Disease-associated circular RNAs: from biology to computational identification. *BioMed Research International*.

[15] Zhong Y., Li X., Li C. (2020). Intracerebral hemorrhage alters circular RNA expression profiles in the rat brain. *American Journal of Translational Research*.

[16] Jiang S., Zhao G., Lu J. (2020). Silencing of circular RNA ANRIL attenuates oxygen-glucose deprivation and reoxygenation-induced injury in human brain microvascular endothelial cells by sponging miR-622. *Biological Research*.

[17] Cao Y., Liu H., Zhang J., Dong Y. (2020). Circular RNA cZNF292 silence alleviates OGD/R-induced injury through up-regulation of miR-22 in rat neural stem cells (NSCs). *Artificial cells, nanomedicine, and biotechnology*.

[18] Jiang L., Li H., Fan Z., Zhao R., Xia Z. (2019). Circular RNA expression profiles in neonatal rats following hypoxic-ischemic brain damage. *International Journal of Molecular Medicine*.

[19] Wang Z., Gerstein M., Snyder M. (2009). RNA-Seq: a revolutionary tool for transcriptomics. *Nature Reviews Genetics*.

[20] Dong X., Zhuang S., Huang Y. (2020). Expression profile of circular RNAs in the peripheral blood of neonates with hypoxic‑ischemic encephalopathy. *Molecular Medicine Reports*.

[21] Suarez-Farinas M., Lowes M. A., Zaba L. C., Krueger J. G. (2010). Evaluation of the psoriasis transcriptome across different studies by gene set enrichment analysis (GSEA). *PLoS One*.

[22] Glazar P. (2014). circBase: a database for circular RNAs. *RNA*.

[23] Xia S., Feng J., Chen K. (2018). CSCD: a database for cancer-specific circular RNAs. *Nucleic Acids Research*.

[24] Agarwal V., Bell G. W., Nam J. W., Bartel D. P. (2015). Predicting effective microRNA target sites in mammalian mRNAs. *Elife*.

[25] Murray D. M. (2019). Biomarkers in neonatal hypoxic-ischemic encephalopathy--review of the literature to date and future directions for research. *Handbook of Clinical Neurology*.

[26] Yasova Barbeau D., Krueger C., Huene M. (2019). Heart rate variability and inflammatory markers in neonates with hypoxic-ischemic encephalopathy. *Physiological Reports*.

[27] Aliefendioglu D., Dogru T., Albayrak M., Dibekmisirlioglu E., Sanli C. (2012). Heart rate variability in neonates with hypoxic ischemic encephalopathy. *Indian Journal of Pediatrics*.

[28] Cai Q., Wang T., Yang W. J., Fen X. (2016). Protective mechanisms of microRNA-27a against oxygen-glucose deprivation-induced injuries in hippocampal neurons. *Neural Regeneration Research*.

[29] Cao Y. H., Li D. G., Xu B. (2016). A microRNA-152 that targets the phosphatase and tensin homolog to inhibit low oxygen induced-apoptosis in human brain microvascular endothelial cells. *Genetics and Molecular Research*.

[30] Irmady K., Jackman K. A., Padow V. A. (2014). Mir-592 regulates the induction and cell death-promoting activity of p75NTR in neuronal ischemic injury. *The Journal of Neuroscience*.

[31] Liu Q., He H., Zeng T., Huang Z., Fan T., Wu Q. (2014). Neural-specific expression of miR-344-3p during mouse embryonic development. *Journal of Molecular Histology*.

[32] Ghafouri-Fard S., Shaterabadi D., Abak A. (2021). An update on the role of miR-379 in human disorders. *Biomedicine & Pharmacotherapy*.

[33] Liu F., McCullough L. D. (2013). Inflammatory responses in hypoxic ischemic encephalopathy. *Acta Pharmacologica Sinica*.

[34] Liu J., Feng Z. C. (2010). Increased umbilical cord plasma Interleukin-1 levels was correlated with adverse outcomes of neonatal hypoxic-ischemic encephalopathy. *Journal of Tropical Pediatrics*.

[35] Mezzasoma L., Antognelli C., Talesa V. N. (2016). Atrial natriuretic peptide down-regulates LPS/ATP-mediated IL-1*β* release by inhibiting NF-kB, NLRP3 inflammasome and caspase-1 activation in THP-1 cells. *Immunologic Research*.

[36] Qu X. Y., Zhang Y. M., Tao L. N. (2019). XingNaoJing injections protect against cerebral ischemia/reperfusion injury and alleviate blood-brain barrier disruption in rats, through an underlying mechanism of NLRP3 inflammasomes suppression. *Chinese Journal of Natural Medicines*.

[37] Li J., Chen J., Mo H. (2016). Minocycline protects against NLRP3 inflammasome-induced inflammation and P53-associated apoptosis in early brain injury after subarachnoid hemorrhage. *Molecular Neurobiology*.

[38] Ham P. B., Raju R. (2017). Mitochondrial function in hypoxic ischemic injury and influence of aging. *Progress in Neurobiology*.

[39] Bai X., Xiong L. L., Fang C. L. (2021). Interleukin 10 plays an important role in neonatal rats with hypoxic-ischemia associated with B-cell lymphoma 2 and endoplasmic reticulum protein 29. *Analytical Cellular Pathology (Amsterdam)*.

[40] Liesz A., Bauer A., Hoheisel J. D., Veltkamp R. (2014). Intracerebral interleukin-10 injection modulates post-ischemic neuroinflammation: an experimental microarray study. *Neuroscience Letters*.

[41] Gu Y., Zhang Y., Bi Y. (2015). Mesenchymal stem cells suppress neuronal apoptosis and decrease IL-10 release via the TLR2/NF*κ*B pathway in rats with hypoxic-ischemic brain damage. *Molecular Brain*.

[42] Mike J. K., Pathipati P., Sheldon R. A., Ferriero D. M. (2021). Changes in arginase isoforms in a murine model of neonatal brain hypoxia- ischemia. *Pediatric Research*.

[43] Petrone A. B., O'Connell G. C., Regier M. D., Chantler P. D., Simpkins J. W., Barr T. L. (2016). The role of arginase 1 in post-stroke immunosuppression and ischemic stroke severity. *Translational Stroke Research*.

[44] Krystofova J., Pathipati P., Russ J., Sheldon A., Ferriero D. (2019). The arginase pathway in neonatal brain hypoxia-ischemia. *Developmental Neuroscience*.

[45] Song X. L., Zhang F. F., Wang W. J. (2020). LncRNA A2M-AS1 lessens the injury of cardiomyocytes caused by hypoxia and reoxygenation via regulating IL1R2. *Genes Genomics*.

[46] Lasda E., Parker R. (2014). Circular RNAs: diversity of form and function. *RNA*.

[47] Bhalala O. G., Srikanth M., Kessler J. A. (2013). The emerging roles of microRNAs in CNS injuries. *Nature Reviews. Neurology*.

[48] Paul R., Bapat P., Deogharkar A. (2021). MiR-592 activates the mTOR kinase, ERK1/ERK2 kinase signaling and imparts neuronal differentiation signature characteristic of group 4 medulloblastoma. *Human Molecular Genetics*.

[49] Gao S., Chen J., Wang Y. (2018). MiR-592 suppresses the development of glioma by regulating Rho-associated protein kinase. *Neuroreport*.

[50] Peng T., Zhou L., Qi H., Wang G., Luan Y., Zuo L. (2017). Retracted - MiR-592 functions as a tumor suppressor in glioma by targeting IGFBP2. *Tumour Biology*.

